# Extensive Iliocaval Deep Vein Thrombosis Provoked by Coronavirus Disease 2019 (COVID-19) in the Setting of an Inferior Vena Cava Filter

**DOI:** 10.7759/cureus.51873

**Published:** 2024-01-08

**Authors:** Anisa Raidah, Nolberto Jaramillo, Katherine F Pradas, Anantha Ramanathan

**Affiliations:** 1 Surgery, New York Institute of Technology College of Osteopathic Medicine, Old Westbury, USA; 2 Surgery, Nassau University Medical Center, East Meadow, USA; 3 Surgery, Stony Brook University, Stony Brook, USA

**Keywords:** covid-19, inferior vena cava thrombosis, sars-cov-2, deep vein thrombosis (dvt), ivc filter complication

## Abstract

Thrombotic events are well-known complications of coronavirus disease 2019 (COVID-19). Inferior vena cava filters (IVCF) are devices used to prevent pulmonary embolism (PE) and also increase the risk of thrombotic complications. Here, we describe the case of a 38-year-old female with extensive bilateral lower extremity deep vein thrombosis (DVT) and thrombosis of the infrarenal inferior vena cava (IVC) in the setting of an IVCF and symptomatic COVID-19. The IVCF had been placed a few months prior for a left femoral DVT and PE after spinal surgery. This patient was treated with pharmacomechanical thrombectomy and ultrasound-assisted thrombolysis followed by angioplasty and anticoagulation. The IVCF was retrieved after confirming there was no residual DVT when the patient recovered from COVID-19 infection. This case of a rare combination of IVCF-related thrombosis secondary to COVID-19 highlights the potential pitfalls of IVCF in this situation.

## Introduction

The severe acute respiratory syndrome coronavirus 2 (SARS-CoV-2) is a virus responsible for the coronavirus disease 2019 (COVID-19) pandemic. Thrombosis has been found in increased frequency in COVID-19 patients including arterial (ischemic strokes) and venous (deep vein thrombosis (DVT), upper extremity thrombosis, pulmonary embolism (PE)) [1]. The underlying mechanism can be understood through the three components of Virchow's triad: venous stasis, endothelial injury, and hypercoagulability. Venous stasis may occur due to hospitalization and increased bed rest in patients afflicted with COVID-19. Endothelial injury may occur from viral tropism for angiotensin-converting enzyme 2 (ACE-2) receptors on endothelial cells leading to cellular invasion and increased angiogenesis. It may also be due to the immune-mediated release of inflammatory cytokines and acute-phase reactants as well as the activation of complement pathways. Hypercoagulability is likely due to the increased expression of prothrombotic factors associated with SARS-CoV-2 infection, including fibrinogen, D-dimer, factor VIII, and von Willebrand factor [2]. An inferior vena cava filter (IVCF) is a mechanical device that reduces the long-term risk of PE by preventing thromboemboli from reaching the heart. These devices are commonly used in the setting of DVT or PE when contraindications to traditional medical therapy exist [3]. Additionally, they may be placed prophylactically [4]. They are associated with an increased risk of inferior vena cava (IVC) thrombosis and lower extremity DVT as compared to anticoagulation alone [3]. They can be placed through endovascular techniques to trap venous emboli from the lower extremity preventing PE [5]. We present the case of a patient with a rare combination of an IVCF and COVID-19 infection who presented with extensive iliofemoral and IVC thrombosis.

## Case presentation

**Figure 1:**
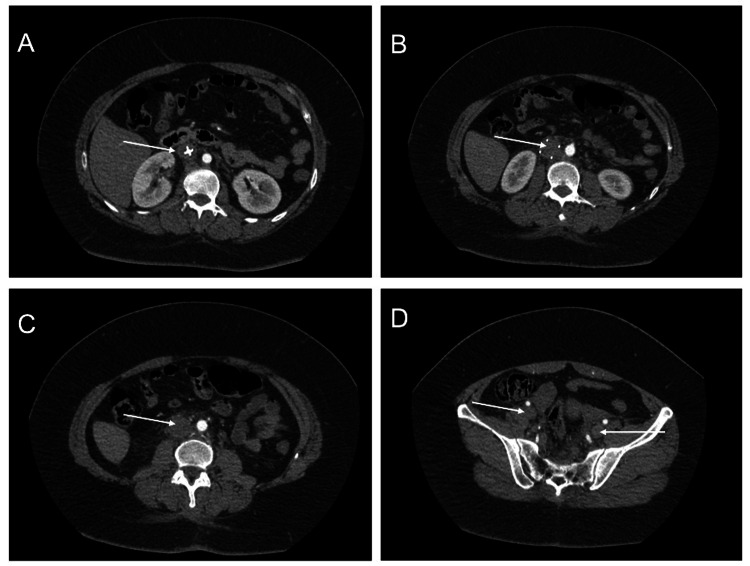
CT angiography demonstrating IVCF thrombosis extending down to the bilateral iliac veins. A) IVCF head. B) Thrombosed IVCF. C) Thrombosis of IVC. D) Thrombosis of bilateral iliac veins. CT: computed tomography; IVCF: inferior vena cava filter

A 38-year-old female presented to the emergency department with left lower extremity pain and erythema for one day that worsened overnight. She stated that this pain was similar to when she was diagnosed with a left l­eg DVT and was worse with movement and weight bearing. Review of her medical history revealed hyperlipidemia, hypertension, type 2 diabetes mellitus, anemia, and an accident requiring lumbar laminectomy and fusion of L4-S1 five months prior. Following this spinal surgery, she had a left femoral DVT and PE, which resulted in the placement of an IVCF. She was not started on anticoagulation due to the spinal surgery. Her husband had a symptomatic COVID-19 infection one week prior. She denied any pertinent family or social history, numbness, tingling, back pain, chest pain, or shortness of breath. Her vital signs were stable at the time, but she later required supplemental oxygen. Physical examination was remarkable for an overweight woman with bilateral lower extremity swelling worse on the left side as well as erythema and mottled skin over the left lower extremity. An initial computed tomography (CT) scan of the thorax showed bilateral ground-glass opacities in the lungs consistent with COVID-19 infection. Viral testing was positive for SARS-CoV-2 infection. Duplex ultrasound of the left lower extremity showed distention and complete non-compressibility without color flow or Doppler signals in the femoral and popliteal veins. CT angiography showed IVC distension, pericaval edema, and fat stranding. There was also a lack of opacification of the infrarenal IVC and iliac veins bilaterally (Figure 1).

CT venography demonstrated a filling defect in the IVC. Thrombophilia screening was negative. The patient was admitted to the hospital and managed with pain control, anticoagulation with enoxaparin, and intravenous antibiotics. On admission to the medicine floors, she required supplemental oxygen and subsequently underwent pharmacomechanical thrombectomy and ultrasound-assisted thrombolysis followed by angioplasty. This was accomplished using the AngioJet™, ZelanteDVT™, and EKOS™ endovascular system (Boston Scientific, Marlborough, Massachusetts, United States). The patient was discharged on oral apixaban which was continued indefinitely. Two months later, a venogram demonstrated residual stenosis of the right common iliac vein which was managed with angioplasty. A stent was not used due to a concern for thrombosis. On subsequent follow-up three months later, the patient was fully recovered from COVID-19 infection, and confirmatory studies revealed no residual DVT. This prompted the retrieval of the IVCF which was performed without complications.

## Discussion

Our patient had extensive iliocaval venous thrombosis in the unique setting of COVID-19 infection and an IVCF placed due to her history of spinal surgery. She was managed with catheter-directed thrombolysis/thrombectomy, angioplasty, and lifelong anticoagulation.

Although commonly used for DVT prophylaxis, anticoagulation increases the risk of bleeding and is contraindicated in many scenarios. Absolute contraindications include coagulation defects, severe thrombocytopenia, recent intracerebral hemorrhage, cerebral lesions with a high risk of bleeding, urgent surgery, and uncontrollable bleeding. Prophylactic indications for IVCF placement include trauma and bariatric surgery although this is controversial [3]. The DVT risk with IVCF is increased as was demonstrated in a study by Decousus et al. where the risk of recurrent DVT in IVCF patients was doubled at two years when compared to a group managed with anticoagulation alone [6]. The IVC is the most common location with a mortality rate twice as high as that of a DVT. The most common cause of IVC thrombosis is the presence of an unretrieved IVCF in the absence of a congenital anomaly [7]. Thus, in patients with IVCF, it is important to closely monitor for the possibility of removal and other complications [5]. Anticoagulation should be continued and the filter removed as soon as there is no longer a contraindication to anticoagulants. The United States Food and Drug Administration guidelines recommend removal of an IVCF within 29-54 days of implantation or as soon as there is no risk of PE [8]. The complications associated with unretrieved IVCF increase in frequency as the filter dwell time increases. These include caval thrombosis, caval wall penetration, migration or fracture of the filter, and an increased risk for DVT. Difficulty in retrieving the filter can also arise due to abnormal filter position or embedding of the filter components into the IVC wall due to endothelization of its components [4].

COVID-19 likely increased our patient's risk of thrombosis. In one study of intensive care unit patients hospitalized due to COVID-19, venous thromboembolisms including DVT and PE were confirmed in 27% of patients, while arterial thrombotic events were reported in 3.7% of patients [9]. In a similar study among critically ill ICU patients with COVID-19, the rate of thrombotic complications was 31% [10]. In non-hospitalized COVID-19 patients, the thrombotic complication rate is unknown; however, a case series has been reported on six non-hospitalized COVID-19 patients with PE on presentation who lacked critical illness or risk factors for DVT [11].

DVT in a patient with COVID-19 infection and IVCF is rare, with few cases reported in the literature. In one case of a patient with multiple pelvic bone fractures, IVCF, and COVID-19 infection, extensive IVC thrombosis was found. Prophylactic doses of heparin could not prevent this complication; however, initiation of rivaroxaban led to complete resolution over weeks [8].

The interplay between the increased risk of thrombosis with COVID-19 infection and IVCF in this patient may have led to this complication. Thus, in patients with an IVCF and this viral infection, careful consideration of the risk of thrombosis should be made when selecting a strategy for DVT prophylaxis.

## Conclusions

IVCF placement in COVID-19 patients requires careful consideration due to the increased risk of severe thrombotic complications. Also, retrieval of an IVCF should be done as soon as possible. Moreover, IVCF carry a risk of harm that must be considered before placing them in patients.
